# A 3D Capillary-Driven Multi-Micropore Membrane-Based Trigger Valve for Multi-Step Biochemical Reaction

**DOI:** 10.3390/bios13010026

**Published:** 2022-12-26

**Authors:** Yijun Zhang, Yuang Li, Xiaofeng Luan, Xin Li, Jiahong Jiang, Yuanyuan Fan, Mingxiao Li, Chengjun Huang, Lingqian Zhang, Yang Zhao

**Affiliations:** 1 Institute of Microelectronics of the Chinese Academy of Sciences, Beijing 100029, China; 2 University of Chinese Academy of Sciences, Beijing 100049, China

**Keywords:** 3D trigger valve, microporous membrane, capillary-driven, gating threshold, POCT

## Abstract

Point-of-care testing (POCT) techniques based on microfluidic devices enabled rapid and accurate tests on-site, playing an increasingly important role in public health. As the critical component of capillary-driven microfluidic devices for POCT use, the capillary microfluidic valve could schedule multi-step biochemical operations, potentially being used for broader complex POCT tasks. However, owing to the reciprocal relationship between the capillary force and aperture in single-pore microchannels, it was challenging to achieve a high gating threshold and high operable liquid volume simultaneously with existing 2D capillary trigger valves. This paper proposed a 3D capillary-driven multi-microporous membrane-based trigger valve to address the issue. Taking advantage of the high gating threshold determined by micropores and the self-driven capillary channel, a 3D trigger valve composed of a microporous membrane for valving and a wedge-shaped capillary channel for flow pumping was implemented. Utilizing the capillary pinning effect of the multi-micropore membrane, the liquid above the membrane could be triggered by putting the drainage agent into the wedge-shaped capillary channel to wet the underside of the membrane, and it could also be cut off by taking away the agent. After theoretical analysis and performance characterizations, the 3D trigger valve performed a high gating threshold (above 1000 Pa) and high trigger efficiency with an operable liquid volume above 150 μL and a trigger-to-drain time below 6 s. Furthermore, the retention and trigger states of the valve could be switched for repeatable triggering for three cycles within 5 min. Finally, the microbead-based immunoreaction and live cell staining applications verified the valve’s ability to perform multi-step operations. The above results showed that the proposed 3D trigger valve could be expected to play a part in wide-ranging POCT application scenarios.

## 1. Introduction

Microfluidic techniques have shown great potential for point-of-care testing (POCT) applications owing to their advantages of high integration, low reagent usage, and rapid detection [1]. In the past decades, microfluidic devices have been widely used for on-site detection of molecules, cells, pathogens, and microorganisms in different scenarios, such as pregnancy detection, food hygiene detection, and COVID-19 virus detection [2,3,4,5,6,7,8]. In conventional microfluidic systems, various microfluidic handlings of liquid samples, e.g., pumping, stopping, mixing, and washing, are usually required for different purposes, in which external or internal liquid control units, such as mechanical (electro, magnetic, etc.) pumps and valves, were usually required [9]. These units dramatically increased the complexity and cost of the microfluidic system. In order to overcome these disadvantages of conventional microfluidic systems in POCT applications, capillary microfluidic chips were introduced in which liquid sample was handled by natural capillary forces and gravity, without other external driving forces [10]. As one of the typical capillary chips, the paper-based strips [11,12] have been successfully used on various POCT applications, such as pregnancy and glucose tests. However, their lack of accurate control limited the application in multi-step complex reactions.

**Figure 1:**
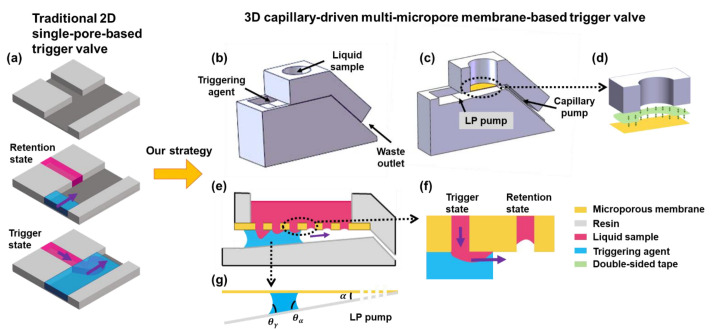
Schematic diagram showing the mechanics and mechanism of the 3D capillary-driven multi-micropore membrane-based trigger valve. (**a**) 3D Schematic and trigger process of traditional 2D single-pore-based trigger valve. (**b**) 3D Schematic of the 3D capillary-driven multi-micropore membrane-based trigger valve. (**c**) Vertical cross-section view of the 3D valve. (**d**) Device assembly schematic. (**e**) Working mechanism of the 3D valve. (**f**) Two states of micropores. (**g**) Laplace pump.

In order to realize a more controllable capillary flow, various capillary valve control units have been reported, including a capillary trigger valve, capillary retention valve, capillary retention burst valve, and delay valve [13,14,15,16]. These capillary valves were designed based on the Laplace pressure difference generated by the changing curvature radius of the liquid meniscus, depending on surface tension, contact angle, liquid consistency, and flow channel shape in 2D microfluidic devices. In these 2D devices, capillary channels and valves mainly included burst pressure valves and trigger valves (Figure 1a) with the triggering agent. Different analytical and numerical models have been reported to study the influence of channel shape, surface characteristics, and fluid characteristics on them [17,18,19,20,21]. However, due to the 2D limitation, the holding time of these valves was usually less than 5 min [22], restricting the time for the user to pre-load the liquid sample and their operational applicability. In order to improve the applicability of the 2D valves, the stair-step liquid-triggered valve has been proposed [23,24,25,26]. The precise channel with a high aspect ratio (i.e., height-to-width ratio) was etched in horizontal and vertical directions to improve valve reliability. The retention time increased to 30 min, attributed to the geometry optimization, but the gating threshold and operating liquid volume were still low (~443 Pa, ~6 μL). Meanwhile, this method had stringent requirements on the aspect ratio of valves [27]. Attempts, such as a high aspect ratio channel or semi-3D valve [28,29,30] design, were proposed to improve the performance of trigger valves for POCT use. However, single-pore microchannel-based trigger valves were limited by the reciprocal relationship between the capillary force and the aperture of the microchannel [31]. To sum up, existing 2D valves had the defects of a low gating threshold, low operable fluid volume, long triggering time, and short retention time. To overcome the drawbacks, we needed a new form of the trigger valve to break the deadlock on the trade-off between high fluid switching ability and high operable liquid volume.

In this paper, we proposed a new 3D capillary-driven multi-micropore membrane-based trigger valve to simultaneously improve the trigger efficiency and operable liquid volume while triggering the valve. With similar trigger structures at the openings of micropores, microporous membranes could achieve a significantly different gating threshold by tuning the dry and wet states. When in the dry state, the gating threshold of the valve could increase to the kPa scale due to the capillary pinning effect of microporous membranes [32], whereas in the wet state, the liquid could drain from millions of micropores on the membrane. Moreover, combining the advantages of the self-driven capillary channel under the membrane for rapidly switching the dry and wet state of the membrane, we proposed the new 3D capillary-driven multi-micropore membrane-based trigger valve, in which the pressure barrier was up to 3500 Pa ( 5 μm micropores, for instance). Meanwhile, the device was triggered within 6 s (duration from trigger to drain of 150 μL liquid, given 5 μm micropores, for instance) and was implemented in multi-step bio-particle immunostaining chain reactions. Furthermore, in the following sections, we characterized the 3D trigger valve’s static performances in different switch states and repeatable performances for cycling switches, respectively, and demonstrated its potential for POCT use in two short applications.

## 2. Design and Experimental Section

### 2.1. Working Principle of the 3D Trigger Valve

The 3D trigger valve was composed of a chamber (loading liquid samples), a microporous membrane (trigger valve), and a wedge-shaped and straight capillary channel (pumps), as shown in Figure 1. The working principle of the valve was described as follows. The triggering agent was first dripped at the entrance of the wedge-shaped capillary channel and was self-driven by capillary force into the channel to infiltrate the microporous membrane. Then, the pore valve was triggered, creating sufficient pressure to overcome the flow resistance. The traction produced by the Laplace pressure pump (LP pump) and capillary pump [33,34] continued driving the liquid and triggering subsequent pore valves.

The microporous membrane was the key element that determined the valve status of open or closed. As shown in Figure 1f, in the retention state, the diagram illustrates the liquid state in the valve. The liquid-gas interface tension exerted an upward force on the liquid column in the micropore due to the surface tension, preventing the liquid sample from fully filling the micropores (it failed to break through the gating threshold of a pore [31]). Conversely, when the triggering agent eliminated the transmembrane pressure of the microporous membrane in the triggering state, as shown in Figure 1f, the liquid-liquid interface formed an outward tension for liquid samples due to the intermolecular force [35]. To improve the trigger efficiency, we designed a wedge-shaped capillary channel under the microporous membrane based on the feeding process of the shorebird effect [36]. When $\theta_{a}<\frac{\pi }{2}+\frac{\alpha }{2}$ and *α* ≥ *θ_a_* − *θ_r_* (where *θ_a_*, *θ_r_*, and *α* were the advancing angle, the receding angle, and the wedge angle, respectively, as shown in Figure 1g), the liquid would fill the corner of the wedge-shaped capillary channel and flow to the slope capillary channel eventually [37,38]. Then, the wedge-shaped capillary channel and microporous membrane returned to the dry state, and liquid samples were retained again. The chamber reverted to a no-leaking vessel and started a new round of reciprocating switching.

With the advantage of the triggering ability of the multi-micropore membrane-based valve and the self-driven ability of the wedge-shaped capillary channel, the combined 3D trigger valve could gain the advantages of befitting the gating threshold and fast response and is suitable for POCT scenarios.

### 2.2. Device Design, Fabrication, and Surface Modification

The overall design idea of the 3D trigger valve followed the basic principles of the mesoscopic scale, which aimed to manipulate liquid samples with a volume of 5 to 200 μL, as is often used in traditional biomedical experiments using 96- or 384-well plates (174927, Thermo Fisher Scientific, Waltham, MA, USA). For that reason, the main structure of the device was designed at the millimeter geometry size. Meanwhile, the feature size of the wedge-shaped capillary channel was 1.5 mm, referring to the capillary length of the water, which is about 2.7 mm [37]. Finally, considering the feature sizes of the single cells and biological microspheres, the 3, 5, and 8 μm micropore diameters were preferred.

The 3D trigger valve was designed as a general-purpose component to achieve multi-step biochemical reaction operations in various POCT scenarios. The requirements of operational capability and duration were referred to the commonly performed biomedical experiments using 96- or 384-well plates. Since the feature sizes of the traditional 2D valves were in micrometers, they were only suitable for the operation and reaction of liquid samples ranging from nanoliter to sub-microliter scale. To meet the requirements in those experiments, we could identify the operable liquid volume of the 3D trigger valve to tens of microliters and the operation duration to tens of seconds per cycle. As shown in Figure 1b, the diameter and height of the cylindrical chamber for the liquid sample were 6 mm and 5 mm, respectively. The wedge-shaped capillary channel’s length, width, and entrance height were 10, 8, and 1.5 mm, respectively. The wedge angle was 3.4°, which had the highest droplet speed because of the balance between the magnitude of the Laplace pressure and its direction [39]. The height and length of the slope capillary channel were designed to be 0.7 mm and 10 mm, respectively. For comparative study, the micropore sizes were chosen as 3 μm, 5 μm, or 8 μm, respectively. Correspondingly, the porosity of the three microporous membranes was 7.1%, 11.8%, and 15.1%, and their thickness was 9 μm, 10 μm, and 15 μm, respectively.

The 3D trigger valve was manufactured by a 3D printer (Lite 300, UnionTech, Shanghai, China) using transparent resin (11122, Mohou, Beijing, China). The polymeric microporous membranes with different pore sizes and porosities were purchased from Lanzhou Heavy Ion Research Facility (HIRFL) lab, CAS of China, where the membranes were fabricated on commercial polycarbonate (PC) membrane using a high-speed heavy ion bombardment and sodium hydroxide etching manufacturing processes.

SEM graphics of microporous membranes with different apertures were taken. We measured the diameter of multiple micropores at different positions, and the average value was obtained (i.e., the pore size of microporous membranes). The number of micropores per unit area was defined as the density of the microporous membrane (*ρ*), and the porosity (*φ*) of the microporous membrane was calculated using the formula (*φ* = *ρπR*^2^).

To improve the hydrophilicity and consistency of the surface characteristics of microporous membranes, we treated them with oxygen plasma for 2 min. The water contact angle of the above-modified surfaces was photographed and measured at room temperature.

### 2.3. Device Performance Evaluation

#### 2.3.1. Evaluation of the Gating Threshold, Seepage Velocity, and Response Time

The whole device evaluation equipment consisted of a horizontal stage, a high-power microscope (HY-300X) with adjustable magnification in both vertical and horizontal directions, a camera (SONY CMOS, 1080P@60fps), a light source, and the 3D trigger valve.

As shown in Figure S1 in the Supplementary Materials, after the device was placed vertically on a horizontal table, the deionized water added a drop of red ink to facilitate the observation used in the characterization experiments. Microporous membranes with diameters of 3 μm, 5 μm, and 8 μm were attached to the side of the transparent tube with a length of 45 cm and an inner diameter of 6 mm. The stained deionized water was slowly injected from the upper side, and the gating thresholds of membranes were calculated by recording the height of the liquid before it broke through the membrane. The seepage velocity of microporous membranes was evaluated by measuring the time for the 1.4 mL liquid to seep through the infiltrated membrane. For the gating threshold, both the untreated and oxygen-plasma-treated microporous membranes were measured. The seepage velocity was measured with the treated microporous membrane. The trigger efficiency of the valve was also accessed by measuring the entire draining time of 150 μL of deionized water. Each experiment with different conditions was repeated three times.

In the transparent tube, the liquid pressure formed by the gravity of the liquid column could be equivalent to the gating threshold of the microporous membranes, which was equal to the product of density, depth, and gravity constant (*P* = *ρgh*). The gating threshold of the microporous membranes could be calculated by substituting the height of the liquid column obtained by the experiment. The porous media seepage theory, dominated by surface molecular forces, could be used to calculate the seepage velocity of microporous membranes. When infiltrating the bottom of microporous membranes, the time (*t*) required for the liquid height to drop from 8 cm to 3 cm (liquid volume *V* = *πR*^2^*h*) was recorded to calculate the average seepage velocity ($u=\frac{V}{t}$).

#### 2.3.2. Reopen and Reclose Performance Evaluation

To evaluate the 3D trigger valve’s performance for the cycling switch, we repeatedly added liquid samples and triggering agents to reopen and reclose the valve and observed the volume of the liquid above the membrane. The pore size of the microporous membrane was 5 μm, and the liquid samples were three groups of 150 μL of deionized water (red ink). We recorded the change of the liquid height using a horizontally positioned microscope to assess the time response of the 3D valve, which showed how quickly the valve was turned on and off. The experiment was repeated three times.

#### 2.3.3. Demonstration of Multi-Step Microfluidic Operations for Bio-Particle Immunostaining

In order to evaluate the capability of multi-step operations, which are usually required in most bio-detection applications, demonstrations of immunostaining of polystyrene microspheres and cells in the proposed 3D trigger valve were performed. The immunostaining process of polystyrene microspheres (PS microspheres, Tianjin BaseLine Chrom Tech Research Centre, Tianjin, China) was as follows. Two 3D microfluidic valves with 5 µm micropores were used as the experimental and control groups. The reaction reagents were 80 µL 150 µg/mL antibody solution (F9512, Sigma-Aldrich, Saint Louis, MO, USA) and 20 µL PS microspheres with a diameter of 7 µm. The difference between the experimental group and the control group was that the former used PS microspheres sealed with the 100 µg/mL IgG antigen (I4506, Sigma-Aldrich, Saint Louis, MO, USA), while the latter used PS microspheres sealed with Bovine Serum Albumin (BSA) (Thermo Fisher, Waltham, MA, USA). After 15 min for the reaction, the Phosphate Buffer Saline (PBS) (GIBCO, Life Technologies Corp., New York, NY, USA) was added to the valve as the liquid sample and triggering agent to wash repeatedly.

The immunostaining process of A549 cells (China Infrastructure of Cell Line Resource) was as follows. First, the 2.5 × 10^5^/mL 50 µL A549 cell suspension was added into the chamber of valves. Then, we added a 2% 50 µL calcein AM solution (Thermo Fisher, Waltham, MA, USA) to the experimental group and 50 µL PBS to the control group. Similarly, we washed with PBS after 15 min.

After the immunostaining reaction, the PS microspheres and A549 cells were observed with the fluorescence microscope and CCD camera. The obtained images under the bright and fluorescence fields were then merged for collaborative observation.

## 3. Results and Discussions

### 3.1. Microfluidic Trigger Valve Geometry and Surface Characterization

**Figure 2:**
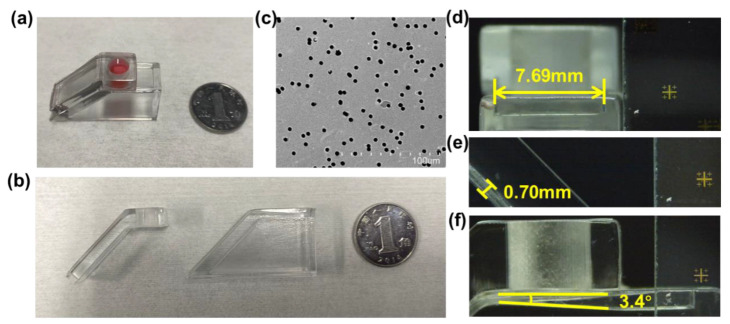
Fabrication of the 3D trigger valve. (**a**) Photograph of the fabricated valve in the assembled state. (**b**) Photograph of the fabricated valve in the disassembled state. (**c**) SEM image of the microporous membrane (pore size = 5 μm, porosity = 11.8%, thickness = 10 μm). (**d**) Microscopic image of the wedge-shaped capillary channel with ~8 mm in width. (**e**) Microscopic image of the slope capillary channel with 0.7 mm in height. (**f**) Microscopic image of the wedge-shaped capillary channel with 3.4° in the wedge angle.

The fabricated 3D trigger valve is characterized in Figure 2, which shows the photograph of the device and the microscopic images of micropores and capillary channels. Figure 2a,b shows the 3D trigger valve in the assembled state and parted state, which consists of a cylindrical chamber for sample loading (filled with red ink), a waste outlet, and different channels for self-driving. Combined with the operable liquid volume commonly used in the laboratory and the size of the 96-well plate, the chamber was designed with a total volume of 150 µL. The 3D valve can handle a 25-fold higher volume of liquid sample than 2D valves [27]. Figure 2c shows a typical SEM image of the microporous membranes with a 5 μm pore size. The microporous membrane was closely bonded to the device structure with the thin double-sided adhesive tape (3M55236, Shanghai, China) (Figure 1d). The thin double-sided tape was cut to the annulus with an inner diameter equal to the diameter of the cylindrical chamber and pasted at the bottom of the cylindrical chamber. The device base was stably placed on a horizontal operating platform, and the bottom of the upper chamber was glued to the device base. Figure 2d–f shows the slope capillary and wedge-shaped capillary channels with a wedge angle of around 3.4° to ensure sufficient Laplacian pressure for the liquid to be completely drained.

**Figure 3:**
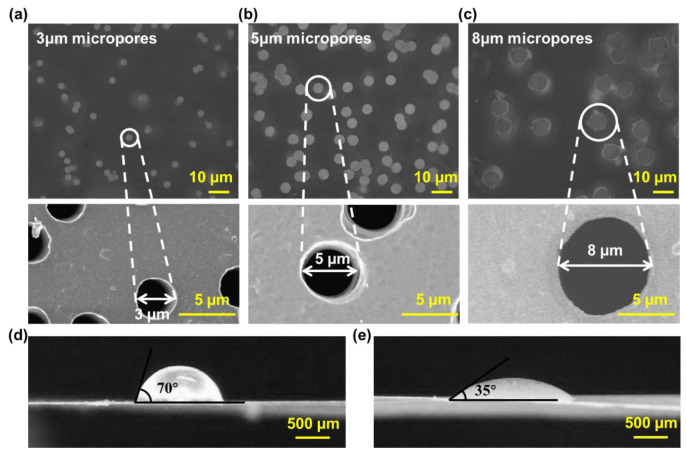
Morphology characterization of microporous membranes. (**a**) The micropores of 3 µm (porosity = 7.1%, thickness = 9 μm). (**b**) The micropores of 5 µm (porosity = 11.8%, thickness = 10 μm). (**c**) The micropores of 8 µm (porosity = 15.1%, thickness = 15 μm). (**d**) The 70° contact angle of the deionized water on the surface of untreated microporous membranes. (**e**) The 35° contact angle of the deionized water on the surface of pretreated microporous membranes.

Figure 3a–c shows the microscopic images of microporous membranes and the corresponding SEM images of a single micropore, indicating that microporous membranes’ pore sizes were etched uniformly to 3 μm, 5 μm, and 8 μm, respectively. Figure 3d,e shows that the contact angle of the untreated and oxygen-plasma-treated membranes was 70° and 35°, respectively. Oxygen plasma treatment could reduce the contact angle by two times and significantly improve the hydrophilicity of microporous membranes.

### 3.2. Retention and Conducting Performance Evaluation

**Figure 4:**
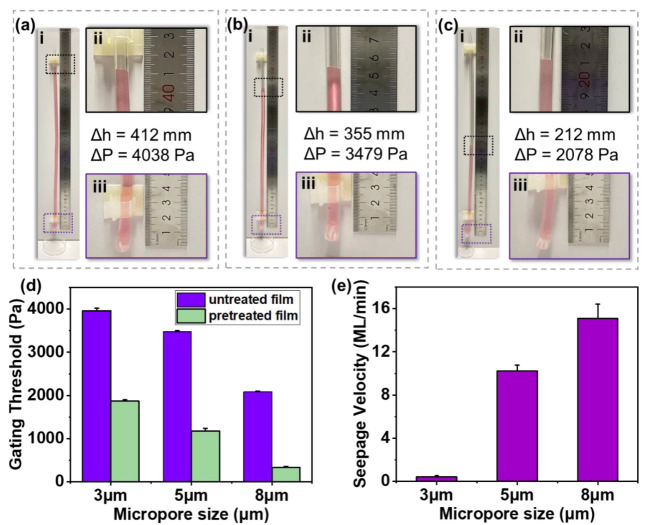
Retention and conducting performances of microporous membranes with the high gating threshold. (**a**) The retention performance of microporous membranes with the 3 µm pore size. (**b**) The retention performance of the microporous membrane with the 5 µm pore size. (**c**) The retention performance of the microporous membrane with the 8 µm pore size. (**d**) Gating thresholds of untreated and pretreated microporous membranes with different pore sizes. (**e**) Seepage velocity of pretreated microporous membranes with different pore sizes.

Since the pore size and porosity would affect the retention performance of the valve, the gating thresholds of the untreated and treated microporous membranes with 3 μm, 5 μm, and 8 μm pore sizes were evaluated. Figure 4a–c shows the tests of untreated membranes, with the gating threshold of 3959.2 Pa for 3 μm pores, 3482.3 Pa for 5 μm pores, and 2080.9 Pa for 8 μm pores. The obtained results showed that with the decrease in the pore size and porosity, the gating threshold increased significantly to require more pressure to trigger, as shown in Figure 3d. According to previous reports, the gating threshold of deionized water in the 2D valve with a 300 μm radius was 420 Pa [31]. However, the gating threshold of the 3D trigger valve was about 3500 Pa due to the dramatic increase in the gating threshold of a single micropore. These advantages ensured the margin and reliability of the 3D trigger valve.

On the other hand, the contact angle would also affect the retention performance of the valve. As shown in Figure 4d, the gating threshold of the untreated microporous membrane with a 5 μm pore size was 3482.3 Pa, while that of the oxygen-plasma-treated microporous membrane with a 5 μm pore size was 1179.0 Pa. Hydrophilic treatment could reduce the gating threshold of microporous membranes by a factor of 3.

Figure 4e shows the tests of oxygen-plasma-treated microporous membranes with different pore sizes, with the seepage velocity of 0.40 mL/min for 3 μm pores, 10.21 mL/min for 5 μm pores, and 15.08 mL/min for 8 μm pores. The seepage velocity was positively correlated with the pore size and porosity. Compared to 2D single-pore trigger valves depending only on the capillary force, the 3D trigger valve had high-density micropores (~1 × 10^6^/cm^2^), which dramatically improved the conducting performance.

### 3.3. Trigger Performance Evaluation

**Figure 5:**
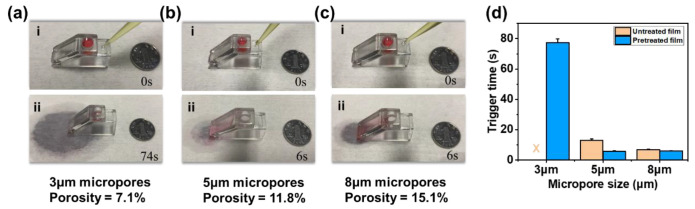
Trigger performances of the 3D trigger valve with different gating thresholds. (**a**) The trigger performance of the valve with the 3 µm pore size. (**b**) The trigger performance of the valve with the 5 µm pore size. (**c**) The trigger performance of the valve with the 8 µm pore size. (**d**) Trigger performance of untreated and pretreated microporous membranes with different pore sizes.

To evaluate the trigger efficiency of the 3D trigger valve, we recorded the trigger times (total draining time of 150 μL of deionized water added with a drop of red ink) with different pore sizes and illustrated them in Figure 5. Trigger efficiency referred to the duration for draining the entire liquid sample, which embodied the conductivity of the 3D valve. In this paper, the cylindrical chamber volume of the device was taken as the standard to evaluate and compare the time required to drain the 150 μL of deionized water totally. The shorter the trigger time, the higher the trigger efficiency. The valve could be triggered for pretreated microporous membranes with a pore size of 5 μm or above within 6 s (MOV S1). However, it was necessary to continuously supply a large volume of trigger agent to achieve complete drainage for microporous membranes with a small pore size (≤3 μm) due to the decrease in the seepage velocity. For untreated microporous membranes, the valve with 3 μm micropores could not be triggered, and the valve with 5 μm micropores increased the trigger time to 13 s, while the valve with 8 μm micropores was unaffected. Therefore, to ensure a sufficient gating threshold and improve the trigger efficiency as much as possible, the oxygen-plasma-treated microporous membrane with a 5 μm aperture was used in the subsequent experiments.

### 3.4. Reopen and Reclosed Evaluation of the Valve

**Figure 6:**
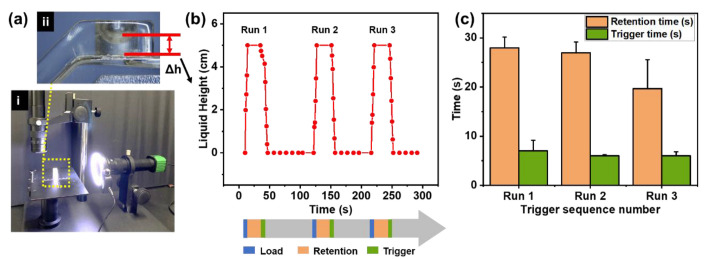
Performance characterization of the 3D trigger valve with repeatable retention-trigger cycle. (**a**) Setup diagram. (**b**) Liquid height variation in three times repeatable retention-trigger cycle. (**c**) The time required for three repetitions to retain and trigger.

The reopening and reclosing ability of the 3D trigger valve with a 5 µm microporous membrane was evaluated, as shown in Figure 6a. The liquid height in the chamber changed in cycles, as shown in Figure 6b and MOV S2. The test results showed that the repetition could be at least three times. In the repeatable retention-trigger cycle, the repetition trigger time was 6 s, and the valve returned to the retention state (drained state) within 60 s, as shown in Figure 6c. Additionally, the 3D trigger valve could be retained without the triggering agent for at least 30 min unless affected by evaporation (Figure S2), while the 2D valve was only cut off for a maximum of 5 min [22]. The results indicated that the 3D trigger valve had the potential for repeatable triggering for multi-step operations.

### 3.5. Triggering Ability for Multi-Step Microfluidic Operations in Bio-Particle Immunostaining Applications

**Figure 7:**
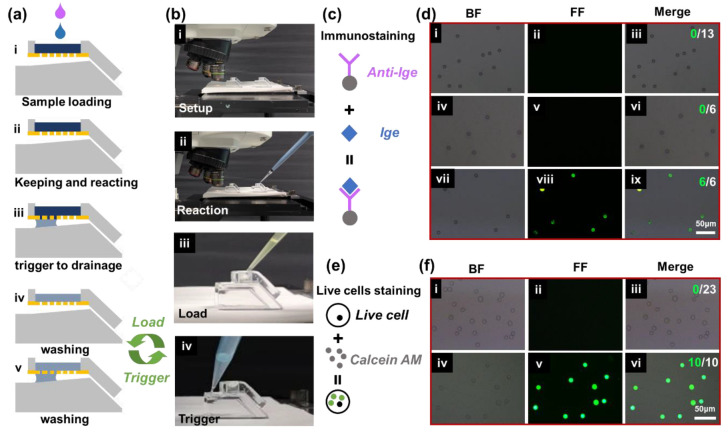
The results of biochemical reactions with multi-step operations. (**a**) The procedure of multi-step chain biochemical reactions. (**b**) Experimental equipment and operation flow chart. (**c**) The principle of immunostaining. (**d**) Staining reaction results of immunostaining. (**e**) The principle of live cell staining. (**f**) Staining reaction results of live cells.

Based on the advantage of repeatable triggering for multi-step operations in the device, we carried out multi-step microfluidic operations and POCT applications. Bio-particle immunostaining refers to the common particle fluorescence phenomenon in bio-detection applications, which often requires multi-cycling washing operations to reduce the interference of background fluorescence. In this paper, the immunostaining experiments of polystyrene microspheres and cells were taken as instances to demonstrate multi-cycling staining and washing operations on the 3D valve. Figure 7a shows the procedure of multi-step microfluidic operations (e.g., sample loading, keeping and reacting, trigger to drainage, and washing), which were performed to mimic the major procedures in a typical biochemical reaction. Two 3D trigger valves with 5 µm micropores were each used as the experimental and the control groups (Figure 7b). The major procedures of PS microsphere and cell line immunostaining are shown in Figure 7c,e. After each step, the bright field microscopic images and fluorescent images were captured and are shown in Figure 7d–f. As shown in Figure 7d, the PS microspheres modified by antigen had no fluorescence phenomenon (Figure 7d-ii). No fluorescence was observed when the antibody was reacted with PS microspheres modified with BSA (Figure 7d-v). The fluorescence phenomenon was only observed in the case of the reaction of antibodies and antigen-modified PS microspheres due to the specific binding (Figure 7d-viii). All antigen-modified PS microspheres in the field showed specific binding.

Figure 7e shows the multi-step procedure of live cell staining. A549 cells had no fluorescence under normal conditions (Figure 7f-ii), while they showed green fluorescence after calcein AM staining (Figure 7f-v). The merged image shown in Figure 7f-vi illustrates that all cells in the field were alive, verifying the precise control and nontoxicity of the valve to live cells. Through the staining experiments of PS microspheres and live cells, we found that the device could accurately manipulate the liquid. Multi-step operations are common procedures for many biological detections, such as pregnancy detection and COVID-19 virus detection, which include the steps of sample loading, reaction on micro-particles, and washing [40]. We demonstrated the applicability of the 3D trigger valve to multi-step operations in microfluidic experiments of micro-particles (within 20 min), which had the potential to be applied to POCT.

Compared with typical 2D trigger valves, the advantages of the 3D valve we propose could be briefly summarized as follows: (1) High gating threshold: 2D valves have a lower gating threshold of 443 Pa [27], while the 3D valve we propose has a higher gating threshold of 3500 Pa (5 μm pore size). The 10-fold higher gating threshold indicates that the device has a higher margin and makes it hard for liquids to leak. (2) High operable liquid volume: 2D valves can only handle 6 μL or an even smaller amount of liquid sample [27], while the 3D valve we proposed can handle a 150 μL liquid sample. The 25-fold larger volume indicates that the device can accommodate more liquid samples. (3) High conductivity: The conductivity of 2D valves is only 10–15 μL/min [1], while the 3D valve we propose can drain the 150 μL of liquid within 6 s after triggering, and the trigger performance is stable, which indicates that the device can handle more liquid samples. (4) Long retention time: 2D valve can only be cut off for a maximum of 5 min [22], while the 3D valve we proposed can keep the cut-off for hours (without considering atmospheric evaporation). It indicates that the device has the capability for temporary sample storage. (5) Multi-cycling reopening and reclosing capability: The 3D valve we proposed can be switched on and off repeatedly more than three times within 5 min, while this parameter was rarely reported in traditional 2D valves. It indicates that the device can be used for multi-step reactions.

## 4. Conclusions

In this study, a new 3D trigger valve was proposed and evaluated based on the theory of Laplace pressure. The valve showed a high gating threshold (~1000 Pa), high trigger efficiency (~6 s), and a large operable liquid volume. The device also showed the capability for repeatable triggering for multi-step operations. Furthermore, the valve was implemented in the staining experiments of PS microspheres and live cells. The capillary-driven 3D trigger valve was implemented for multi-step biochemical reactions, and the obtained results indicated that the device had the potential to be used for POCT applications without an external driving force.

## Data Availability

Not applicable.

## Supplementary Materials

The following supporting information can be downloaded at: https://www.mdpi.com/article/10.3390/bios13010026/s1, Figure S1 shows the device for evaluating the gating threshold. Figure S2 shows the retention performance of the 3D trigger valve. MOV S1 showed the trigger process of the valve with the 5 µm pore size. MOV S2 showed the repeatable retention-trigger cycle of the 3D trigger valve.
