# Supplementary material for: A 3D Capillary-Driven Multi-Micropore Membrane-Based Trigger Valve for Multi-Step Biochemical Reaction
Source: Biosensors (Basel). 2022 Dec 26;13(1):26. doi: 10.3390/bios13010026 (PMC9855807; doi:10.3390/bios13010026)
Supplement: Supplementary file 1 [file biosensors-13-00026-s001.zip › biosensors-2092053-supplementary.pdf]

Supporting information

A 3D Capillary-Driven Multi-Micropore Membrane-Based Trigger Valve for Multi-Step Biochemical Reaction

Yijun Zhang [1,2], Yuang Li [1,2], Xiaofeng Luan [1,2], Xin Li [1,2], Jiahong Jiang [1], Yuanyuan Fan [1], Mingxiao Li [1], Chengjun Huang [1,2], Lingqian Zhang [1,*] and Yang Zhao [1,*]

[1] Institute of Microelectronics of the Chinese Academy of Sciences, Beijing 100029, China
[2] University of Chinese Academy of Sciences, Beijing 100049, China
* Correspondence: zhanglingqian@ime.ac.cn (L.Z.); zhaoyang@ime.ac.cn (Y.Z.)

1. The Device of Evaluating the Gating Threshold

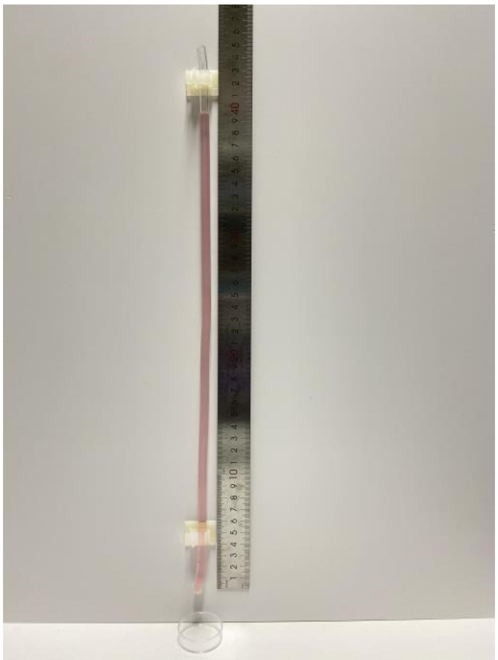

Figure S1. The device of evaluating the gating threshold.

2. Retention Performance of the 3D Microfluidic Valve

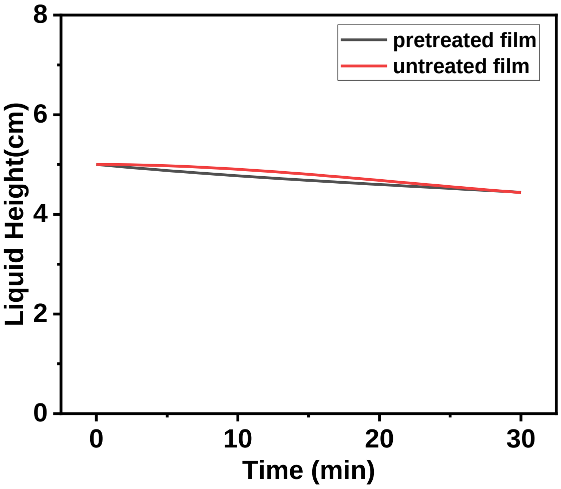

Figure S2. Retention performance of the 3D microfluidic valve.
